# Open-Cellular Alumina Foams with Hierarchical Strut Porosity by Ice Templating: A Thickening Agent Study

**DOI:** 10.3390/ma14051060

**Published:** 2021-02-24

**Authors:** Kathleen Dammler, Katja Schelm, Ulf Betke, Tobias Fey, Michael Scheffler

**Affiliations:** 1 Institute for Materials and Joining Technology, Otto-von-Guericke University Magdeburg, 39106 Magdeburg, Germany; Katja.schelm@gmx.de (K.S.); ulf.betke@ovgu.de (U.B.); m.scheffler@ovgu.de (M.S.); 2 Department of Materials Science and Engineering, Institute of Glass and Ceramics, Friedrich-Alexander Universität Erlangen-Nürnberg (FAU), 90762 Erlangen, Germany; tobias.fey@fau.de; 3 Frontier Research Institute for Materials Science, Nagoya Institute of Technology, Gokiso-cho, Showaku, Nagoya 466-8555, Japan

**Keywords:** foams, replica, ice templating, alumina, freeze casting

## Abstract

Alumina replica foams were manufactured by the Schwartzwalder sponge replication technique and were provided with an additional strut porosity by a freeze-drying/ice-templating step prior to thermal processing. A variety of thickeners in combination with different alumina solid loads in the dispersion used for polyurethane foam template coating were studied. An additional strut porosity as generated by freeze-drying was found to be in the order of ~20%, and the spacings between the strut pores generated by ice-templating were in the range between 20 µm and 32 µm. In spite of the lamellar strut pore structure and a total porosity exceeding 90%, the compressive strength was found to be up to 1.3 MPa. Combining the replica process with freeze-drying proves to be a suitable method to enhance foams with respect to their surface area accessible for active coatings while preserving the advantageous flow properties of the cellular structure. A two-to-threefold object surface-to-object volume ratio of 55 to 77 mm^−1^ was achieved for samples with 30 vol% solid load compared to 26 mm^−1^ for non-freeze-dried samples. The freeze-drying technique allows the control of the proportion and properties of the introduced pores in an uncomplicated and predictable way by adjusting the process parameters. Nevertheless, the present article demonstrates that a suitable thickener in the dispersion used for the Schwartzwalder process is inevitable to obtain ceramic foams with sufficient mechanical strength due to the necessarily increased water content of the ceramic dispersion used for foam manufacturing.

## 1. Introduction

Ceramic foams with an open pore structure are commonly used, e.g., as filters, supports for heterogeneous catalysts, heat exchangers and silencers [1,2,3,4,5]. Often, a high specific surface area and high mechanical stability are required for these applications. The manufacturing is typically carried out by a sponge replication technique, known as the Schwartzwalder process [6], and the resulting ceramic foams possess hollow struts. Those cellular materials own a specific surface area of 1–2 m^2^ g^−1^, which is comparably low [7] for application as catalyst carriers, for instance. The specific surface area has been increased in various studies, for instance, by coating them with a high surface area washcoat [8] or using a porous alumina powder [9].

For those applications where active coatings are a component of the foam strut surface, a higher surface area may be of advantage. As an example, adsorption heat storage or adsorption heat pump materials are addressed [10,11]. Accessibility to the inner struts, a higher specific surface area by a controlled introduction of pores into the strut material or a combination of both may significantly increase the specific surface area, which, in turn, may lead to a higher load with an active coating material, such as zeolites or Metal organic frameworks (MOFs) [11] and thus expand the range of possible applications. A potential processing route to access the inner strut surface and to increase the strut porosity is the combination of the Schwartzwalder process with the freeze-drying technique, as shown in literature for hydroxyapatite-ceramics (with coating of 75 ppi Polyurethane (PU) templates) [12], SiO_2_ ceramics [13] and alumina ceramics (with a coating of 20 ppi PU templates) [14]. Freeze-drying is a common tool [3,15,16,17] to introduce oriented porosity in bulk ceramics [1,18,19], with bioceramics also among them [20].

The feasibility of the combination of the ceramic-foam replica process with the freeze technique was recently demonstrated for alumina foams by Schelm et al. [14]. While the replica process leads to a cellular porosity with pores in the range of the cellular template cells/windows (e.g., for 20 ppi templates in the mm-range), the hollow struts (for 20 ppi in the range of 100 µm) and the remaining bulk porosity after sintering, an additional porosity with lamellar-shaped pores was introduced by a freeze-drying step subsequently after foam template coating. The spacing of the pores and the lamellar thickness after thermal processing was shown to be a function of the cooling rate and the cooling temperature, as the freezing agent water was used [14].

**Figure 1:**
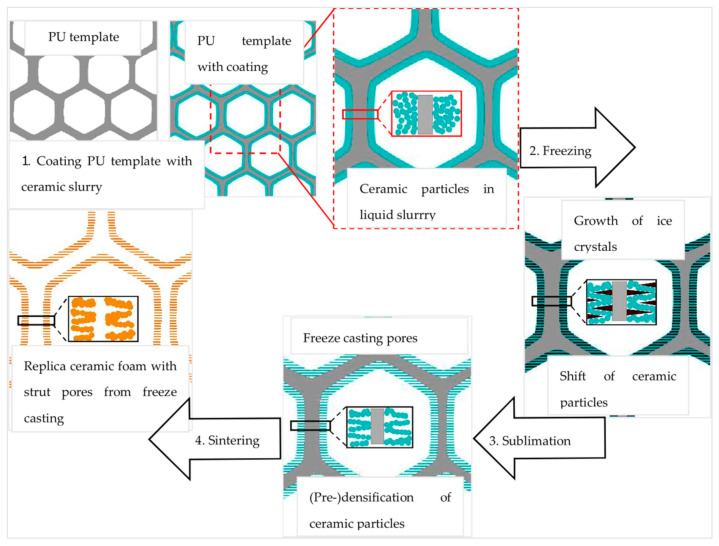
Illustration scheme of the experimental procedure for manufacturing alumina freeze-dried replica foams.

Figure 1 illustrates the combination of both processing methods. In the first step, a polyurethane (PU) sponge is coated with a water-based alumina particle containing dispersion; excess dispersion is removed by gentle squeezing (Figure 1, top left); this is referred to as the Schwartzwalder process, polymer sponge method or replica process [6]. This coating develops the dense stuts with a triangular void, whereas the strut material itself can contain residual porosity (depending on sintering conditions), inside, the struts are hollow as a consequence of the template burnout. In case of freeze-drying, the struts possess additional lamellar pore spacings. During the freeze-drying process, ice crystals form, leaving behind a lamellar shape in the freezing material (Figure 1, center right) [21]; the sublimation (Figure 1, center bottom) is driven by a decrease in pressure and temperature.

The resulting porosity is controllable by the variation of the processing parameters such as the solid content of the coating suspension, the freezing temperature, the amount and type of additives (amongst others sucrose, trehalose, D(+)—trehalose dehydrate, glycerol, ethanol, sodium chloride) [22]. Within the freezing process, control of nucleation of the ice crystals, which occurs at nucleation seeds, and their growth, is important for the pore spacing control [14,15,20,23,24]. Fast cooling rates lead to an increased magnitude of supercooling ahead of the solidifying interface and as a consequence a decrease of the tip radius of the ice crystals, thereby a finer microstructure is achieved [18,21].

Besides water, camphene can be used as a freezing vehicle, due to its ability to form dendritic structures during freezing, and it sublimes at room temperature, which means that no specific sublimation equipment is necessary. Further details can be found elsewhere [25,26,27,28,29,30].

The final step is the thermal processing, which consists of the PU burn out and the sintering, which is carried out after PU burn out or after PU coating, freeze-drying, PU burn out in this specific case (Figure 1, bottom left).

In [14] was stated that there is a conflict between both processing routes: The replica method needs high solid loads to achieve a moderate to high compressive strength of high-porosity materials. In contrast, the freeze-drying process is well controllable with low solid loads. This, in turn, complicates the handling of samples prior to sintering or leads to a non-acceptable low compressive strength. While in our previous work [14] the influence of the freezing temperature (and thus the cooling rate) and the solid load was studied on the porosity and the compressive strength, in this paper we varied the type and amount of the thickening agent as a processing aid in the coating dispersion; the solid load was similar to that in Schelm et al. in ref. [14].

## 2. Materials and Methods

### 2.1. Ceramic Foam Processing

The alumina powder used in this study was CT3000SG with an average particle size of 0.67 µm (Almatis GmbH, Ludwigshafen, Germany). As a deflocculant, Dolapix CE64 (Zschimmer and Schwarz GmbH and Co. KG, Lahnstein, Germany) was added to the powder. In the next step, a dispersion/solution of a thickener, see Table 1, was added and the mixture was stirred with a spatula and subsequently mixed with a planetary centrifugal blender (Model ARE-250, Thinky Corporation, Tokyo, Japan) for 15 min at 2000 rpm. After this mixing step, a polyvinyl alcohol binder (Optapix PA 4G, Zschimmer and Schwarz GmbH and Co. KG, Lahnstein, Germany) was added and the mixing step was repeated with the same mixing duration and rotational speed. Thickening agents used in this work were:

- guar gum, G (Guarkernmehl, Arche Naturprodukte GmbH, Hilden, Germany)
- modified methyl cellulose (wallpaper paste), WP (Spezialkleister, Wilckens Farben GmbH, Glückstadt, Germany)
- methyl cellulose, MC (160,000 g mol^−1^, Carl Roth GmbH&Co. KG, Karlsruhe, Germany) and
- starch (potato) S (sauce binder, Mondamin, Unilever Deutschland GmbH, Hamburg, Germany).

Regarding the different characteristics of the thickeners, a diverse behavior in the ceramic dispersion could be expected, not only in terms of the thickening effect, which is high for guar gum and starch versus low to high for methyl cellulose, but also in terms of the template coating capability (film formation high for starch and methyl cellulose, but not for guar gum). This is likely to be reflected in the results. The properties of the thickeners used are summarized in Table 2.

Thickener formulations from the food and glue industries were used due to their long-term availability and constant quality over several batches of these products and their homogeneous consistency when dispersed/dissolved in water.

As water-based dispersions, two different solid loads with alumina (30 vol% and 40 vol%) were investigated in combination with the thickening agents as shown in Table 2. The compositions of the dispersions are listed in Table 3.

Prior to template coating with the different dispersions, viscosity measurements of these ceramic dispersions were carried out in a plate–plate-viscometer (MCR-301, Anton Paar GmbH, Graz, Austria). The shear stress was measured for shear rates from 0.1 s^−1^ to 100 s^−1^. These measurements were carried out in order to a) to study the influence of the different thickeners on the dispersion formulation and b) to adjust the dispersion viscosity for a proper coating/to ensure reproducible coating conditions. During the viscosity measurements, the measuring system was covered to reduce the water evaporation from the dispersion.

The PU template pieces (15 × 15 × 20) mm^3^ for coating (Koepp Schaum GmbH, Oestrich-Winkel, Germany, 20 ppi) were separately dipped into the dispersions. After withdrawal from the dispersion, they were gently squeezed to remove additional dispersion from the coated foam template and to open still closed-cell windows. The samples were weighted in the wet state to guarantee almost the same weight for all samples(~1.6 g for each coated sample). After coating, the samples were put into a freezing compartment of a household freezer at −20 °C and hold for 24 h to generate the ice crystals responsible for the pore formation within the foam struts. For comparison, a certain number of coated foam templates were dried at ambient conditions (20 °C, atmospheric pressure); this was carried out with 12 to 15 pieces of foam for each type of thickener and 30 vol% as well 40 vol% particle load. After freezing the foams were placed in a freeze-dryer (Alpha 1–4 LDPlus, BETA, 1–16 LMC-2, Martin Christ Gefriertrocknungsanlagen GmbH, Osterode am Harz, Germany), the condenser temperature was set to −55 °C and a pressure of 0.1 mbar was adjusted. Freeze-drying was carried out for 24 h.

After freeze processing, thermal treatment processes were carried out. In a first heating step, the template was removed at 400 °C in air in a circulating air furnace (KU 40/04/A, THERMCONCEPT Dr. Fischer GmbH, Bremen, Germany) with a heating rate of 3 K min^−1^ and a three hours dwell time. In the last step, the foams were sintered in a chamber furnace (Modell HTL 04/18, THERMCONCEPT Dr. Fischer GmbH and Co. KG, Bremen, Germany) at 1650 °C in air with a dwelling time of three hours at 600 °C and at peak temperature; heating and cooling rates were set to 3 K min^−1^. Weight analysis after freeze-drying and sintering showed for each piece of foams a weight of 1.2 g for the templates coated with the 30 vol% dispersion and 1.3 g for those coated with the 40 vol% dispersion. This, however, makes a comparison of resulting properties easier.

In summary, sixteen different foam series with approximately 12 samples of each series, eight series without and eight series with an additional freeze-drying step, were manufactured.

### 2.2. Micro- and Macrostructure Analysis

The strut porosity of the ceramic foams was determined by the Archimedes’ principle according to DIN EN ISO 1183 [32]. A hydrostatic scale (Cubis, Sartorius, Göttingen, Germany) with an accuracy of 0.01 mg for weight and buoyancy measurements for the open strut porosity determination was used, see also Schelm et al. [14].

The determination of the total foam porosity was carried out by measuring the geometric volume of a sample (total rectangular bloc foam volume) of 2.5–3.2 cm^3^ with a caliper and the weight of the samples after sintering (new Classic MF Model: ML204/01, Mettler Toledo Intl. Inc, Columbus, OH, USA).

For scanning in an X-ray microcomputertomograph (µ-CT) selected foams were fixed separately on the sample holder of the µ-CT (Nanotom S, GE Sensing and Inspection Technologies, Wunstorf, Germany). The voltage was adjusted to 50 kV, the tube current was set to 110 µA, and the distance between the sample holder and X-ray tube and detector was defined to obtain a voxel size of 3.5 µm^3^ for scanning a small volume element of a foam sample (approx. 3–4 mm^3^ in size) with high resolution. The reconstruction of the data was performed with the software package Phoenix Datos |X 2.0 (GE Sensing and Inspection Technologies, Wunstorf, Germany). The lamellar pore spacing distribution and the material lamellae thickness distribution were determined from the µ-CT reconstruction data with the software CT Analyzer 1.18 (Skyscan/Bruker microCT, Kontich, Belgium). The average pore spacing and material lamellae thickness was determined from the obtained histograms by fitting one (or a set of) Gauss function(s). More details on the calculation procedure are available in Betke et al. and Schelm et al. [14,33].

Scanning electron micrographs were generated with a scanning electron microscope (SEM, XL30 ESEM-FEG, FEI/Phillips, Hillsboro, OR, USA). Volume elements, as well as embedded and ground cross-sections of the samples, were analyzed. The volume elements of samples were mounted on the sample holder with carbon paste (PLANO Leit C, Plano GmbH, Wetzlar, Germany), resin-embedded samples were glued on the sample holder of the SEM with silver paste, both kinds of samples were sputter-coated with gold.

### 2.3. Compressive Strength Measurements

The compressive strength was measured with a TIRATEST 2825 (TIRA GmbH, Schalkau, Germany). For the measurement of the samples thin squared pieces of cardboard were placed under and over the to-be-measured foam pieces of the size (2.5–3.2 cm^3^) to guarantee a proper force transfer to the entire area of the foam. The cross-head speed used was 2 mm min^−1^; ten species of each sample series were measured and the values were averaged.

## 3. Results and Discussion

Al_2_O_3_ replica foams with increased open strut porosity were manufactured, as already reported previously for varying the freezing temperature [14]. The foam microstructure and, as a consequence, the mechanical properties are influenced—amongst others—by the thickening agent as well as the solid content of the ceramic dispersion.

### 3.1. Rheological Behavior of Ceramic Dispersions

**Figure 2:**
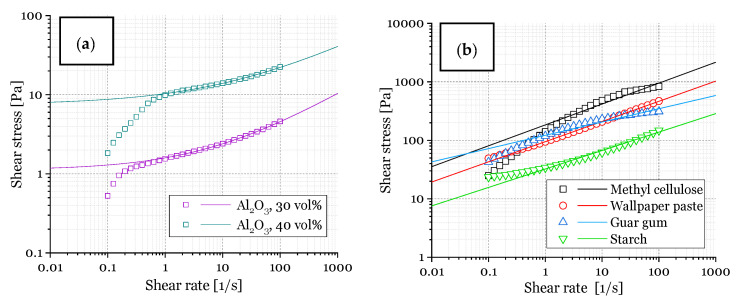
Shear stress vs. shear rate: (**a**) of alumina dispersions (30 vol% and 40 vol%) in water with the additives binder and deflocculant (solid lines represent a fit via the Herschel–Bulkley-model); (**b**) thickening agents dissolved in water (solid lines represent a fit via the Power-Law model), see Table 1.

Viscosity measurements were carried out to obtain alumina dispersions with rheological properties suitable for PU template coating; this means a shear-thinning behavior and the desired viscosity at a given shear rate are necessary [33,34]. Another issue is the water content necessary for freeze-drying [14]. Figure 2a shows the shear stress-shear rate curves of the alumina/water system (with additives: binder, deflocculant, ref. to Table 3) with 30 vol% alumina and 40 vol% alumina, which are suitable for freeze-drying driven generation of pores, as shown in previous works [14,18]. In addition, the flow behavior for the plain thickening agent/water system (without additions, Figure 2b) was analyzed. It was shown before in literature that the thickening agents chosen (forming a gel–network with water) are commonly used in ceramic processing (for example starch [35,36,37,38,39,40,41,42,43,44,45,46,47], guar gum [14,48], methyl cellulose [49,50]). Starch is well known from starch consolidation casting to act as a thickening agent and pore former.

The flow curves of the investigated alumina dispersions could not be approximated adequately with the commonly used Bingham model, which describes a Newtonian flow behavior above a given yield stress τ_0_ [51]. However, the shear stress–shear rate plots of the investigated alumina–water dispersions (Figure 2a) were fitted with the Herschel–Bulkley model (Equation (1) [52]. This model combines a yield stress τ_0_ with a shear-thinning flow behavior for stress values exceeding τ_0_, see Equation (1).

τ = τ_0_ + K γ˙^n^ (1)

The measured shear stress–shear rate data were fitted starting at a shear rate of 1 s^−1^, due to the wall slip effect present for the investigated dispersions [53,54], which would falsify the approximation, as described in the literature [55]. The alumina dispersions show a yield stress τ_0_ that is increasing for an increasing alumina solid content (1.2 Pa for 30 vol% Al_2_O_3_; 7.5 Pa for 40 vol% Al_2_O_3_, 15.22 Pa for 43 vol% Al_2_O_3_ [49]).

In contrast, the flow curves of the thickening agent–water mixtures show a flow behavior in accordance with the Power Law, which describes a Non–Newtonian behavior without a yield stress τ_0_ (= 0) [56]. All dispersions show a shear-thinning behavior, as indicated by the flow index n < 1 (0.43 for 30 vol% Al_2_O_3_; 0.36 for 40 vol% Al_2_O_3_; 0.36 for MC; 0.35 for WP; 0.23 for G; 0.32 for S) [14,49], which is important for a successful coating (especially entering of dispersion into template pores) of PU templates during the sponge replication process [33,57,58].

**Figure 3:**
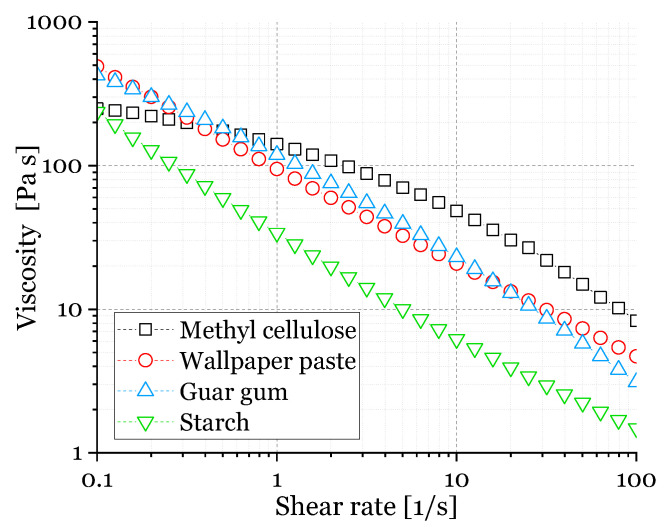
Viscosity vs. shear rate of ceramic dispersions with 30 vol.% solid load for coating of Polyurethane (PU) templates.

Figure 3 represents the viscosity-shear rate plots of the ceramic dispersions used in this study, which possess also a shear-thinning behavior, as found in the literature, especially for dispersions containing methyl cellulose and guar gum [48,50,59,60]. As the shear rate, which is applied to the dispersions by squeezing the PU template during coating is unknown, the viscosity at a shear rate of 100 s^−1^ was selected for further discussion [50]. This value is in the range of shear rates in typical extrusion processes which are 10–1000 s^−1^, ref. [54]. As shown in Figure 3, the viscosity at a shear rate of 100 s^−1^ (η_100_) for the dispersion containing methylcellulose (η_100_ = 12.9 Pa s) is approximately one order of magnitude higher compared to dispersions containing guar gum (η_100_ = 1.07 Pa s) and starch (η_100_ = 1.35 Pa s), the viscosity with wallpaper paste (η_100_ = 3.43 Pa s) is between those values.

Generally, the viscosity decreases for an increasing shear rate for all dispersions, and the flow behavior is in a range suitable for the manufacturing of ceramic foams by the replica technique [33,34,58,59].

### 3.2. Macro- and Microstructure of Sintered Foams

The total foam porosity, as determined from the geometrical dimensions and the foam’s weight, was approximately 90% for all samples. Discrete values are discussed below.

**Figure 4:**
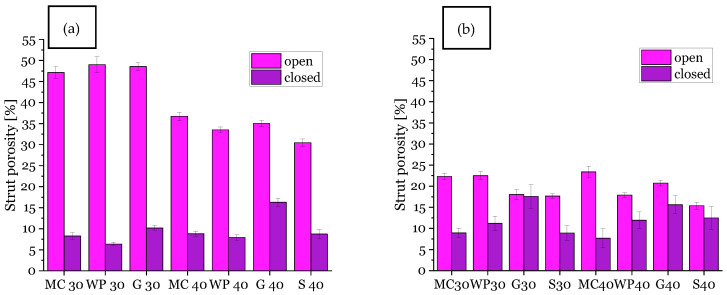
Open and closed strut porosity of samples with 30 vol% (30) and 40 vol% (40) solid load in ceramic dispersion, (**a**): with additional strut porosity from freeze-drying, (**b**): without additional strut pores/conventionally dried samples; MC = methylcellulose, WP = wallpaper paste, G = guar gum, S = Starch.

The open and closed strut porosity results as measured with the water immersion test (Archimedes method DIN EN 623-2, [9,14]) are shown in Figure 4.

The total strut porosity of the replica foams with additional strut pores from freeze-drying is increased compared to foams dried at ambient conditions. The reason for this is the evaporation of water during the drying at ambient conditions in contrast to keeping water inside of the ceramic coating via a transformation into ice crystals and subsequent sublimation converting the ice crystals into lamellar pores. This process is directional and tends to particle movement (pre-densification), as the ice crystals expand during their formation—that is the reason for lamellar-shaped pores in the struts of the foams [18,21] and partly a moderate compressive strength, which will be discussed below.

**Figure 5:**
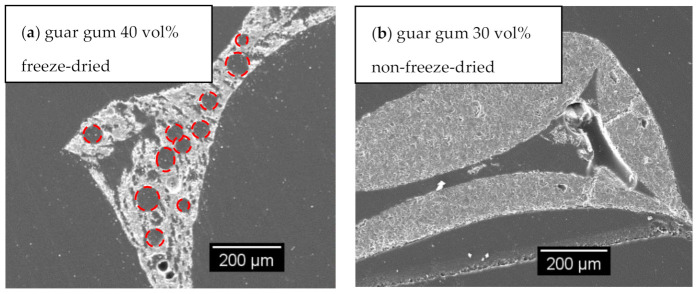
SEM micrographs of sintered foam samples (**a**) sample with guar gum, 40 vol.-% solid content and freeze-drying pores (holes from air bubbles marked with red circles), (**b**) sample with guar gum, 30 vol.-% solid content, conventionally dried at room temperature (pls. refer: [14]).

In particular, the open strut porosity increased by applying the freeze casting technique for replica foams with 30 vol% alumina content compared to samples with 40 vol% alumina dispersion content, reaching an average strut porosity of 48% (for comparison: 20% for samples dried at ambient conditions/non-freeze-drying). This indicates a complete passage of the freeze casting lamellae through the foam struts. If that would not be the case, closed porosity would increase instead (as shown in SEM micrograph for samples with guar gum, Figure 5a and increased closed strut porosity, Figure 4a from entrapped air).

**Figure 6:**
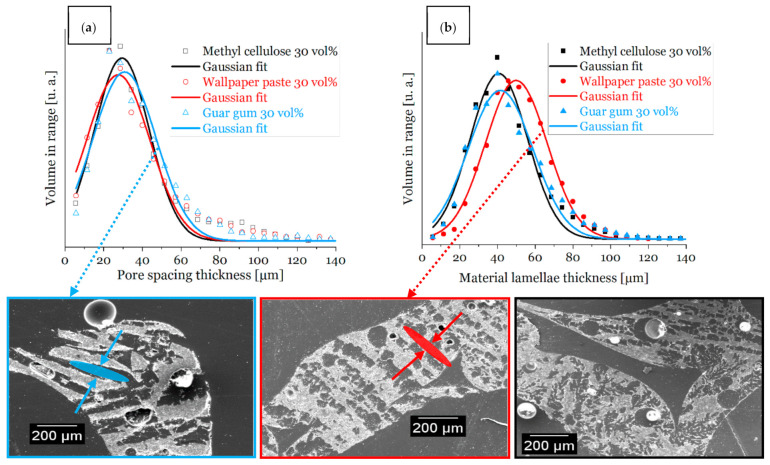
Distributions of the width of material lamella and pore spacings of sintered foam samples from dispersions with 30 vol% solid load measured by µ-CT and SEM images; (**a**): distribution of the pore spacing (**a**) and material lamella thickness (**b**) of sample struts, respectively; (bottom): SEM micrographs of cross-sections of sintered foams; pore spacings and material lamellae exemplary marked with arrows; MC = methylcellulose, WP = wallpaper paste, G = guar gum.

The SEM micrographs are also in good agreement with these results—a high number of the pore spacings are open to the outside of the strut surface in foams with a decreased solid content of the coating slurry (Figure 6).

The widths of the material lamellae and the pore spacing were calculated from µ-CT data collected on one selected sample per sample series according to the procedure described in [14,33].

**Figure 7:**
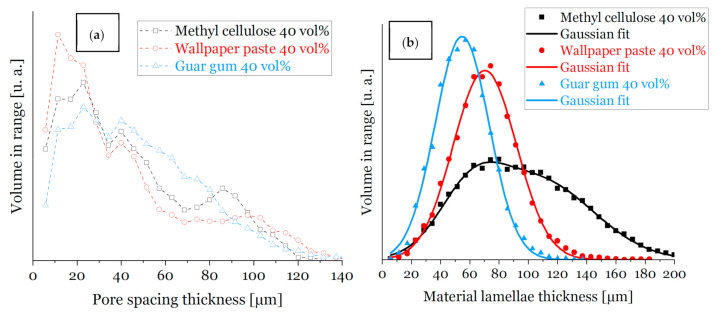
Distributions of the width of material lamella and pore spacings of sintered foam samples from dispersions with 40 vol.-% solid load measured by µ-CT; distribution of the (**a**) pore spacing thickness and (**b**) material lamella thickness of sample struts from sintered foam samples, respectively.Freeze-dried replica foams with starch flour as a thickening agent made from 30 vol% alumina dispersions broke during PU burnout; their stability was insufficient, most likely due to the high amount of lamellar pores within the foam structure.

For a dispersion solid content of 30 vol% (Figure 6) the material lamella thickness for all thickening agents is fairly in the same range (between 43 µm and 53 µm). For an increased solid content in the ceramic dispersion (40 vol%, Figure 7) the distribution of the material lamella thickness is broader and more varying, those values are in a range between 53 µm and 95 µm. This is a consequence of a higher amount of ceramic particles in the dispersion [3,14]. As expected, the thickness of the pore spacing shows an inverse behavior and increases for lower solid content of the ceramic dispersion (for 30 vol% solid load between 29 µm and 33 µm), due to increased water content, and, consequently, a broader ice front during freezing [3,14].

**Figure 8:**
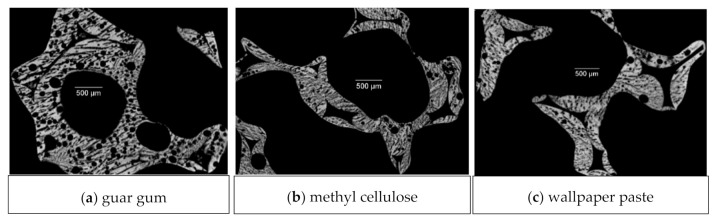
µCT-Images of samples with 40 vol% solid content (**a**) guar gum, (**b**) methylcellulose, (**c**) wallpaper paste.3.2. Microstructure of Dried Samples (Coating Quality).

For samples made of 40 vol% alumina dispersions (Figure 7 and Figure 8), the lamellae pore thickness decreases in comparison to samples from dispersions with a reduced solid load of 30 vol%. There is one exception: samples with guar gum as a thickener, justified by round voids, ref. Figure 5 and Figure 8a), which is in good accordance with the results of previous work [14].

**Figure 9:**
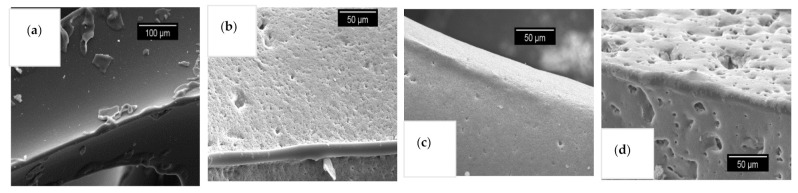
SEM micrographs of (**a**) original Polyurethane (PU) template, (**b**–**d**) coated PU templates with ceramic dispersions with 30 vol% solid load and different thickeners (**b**) Starch, (**c**) Methyl cellulose, (**d**) Wallpaper paste, dried at room temperature (before sintering—PU-template is still intact).

A smaller lamella pore thickness leads in general to an increase of the compressive strength [14,22], as shown within Section 3.2, and in Figure 9. SEM micrographs (Figure 6) demonstrate the lamellar shape of the pores obtained from freeze casting and for all thickening agents the direction of single lamellar pores is non-oriented (Figure 6) originating in non-directional freezing, which should be a benefit for compressive strength of the as-produced samples with additional strut pores.

Summarizing this, it can be stated that the amount, respectively the increased fraction of open strut porosity according to the water immersion test, (ref. Figure 4) and size of the pores, increased for a decreased solid content (30 vol% alumina dispersions). These findings are in accordance with the literature [14,61]. The temperature gradient, or freezing velocity, respectively, was constant during processing within this work, therefore differences in the thickness of the material lamellae/pore spacings originate in the solid–water ratio and the thickening agent also with respect to the flow behavior, as described in the following section (gas bubbles).

Another issue is bubble-shaped voids within the microstructure of replica foams with pores from freeze-drying—especially for guar gum as a thickener: Figure 5a shows a cross-section of a replica foam strut with additional porosity from freeze-drying. Visible voids in Figure 5 and Figure 8a originate from air bubbles during the template coating. These spherical pores are most likely caused by gas bubbles being present in the dispersion. For the dispersions containing 40 vol solids, which have a significantly higher viscosity and yield stress compared to the dispersions with 30 vol% solid loading, the gas bubbles can not escape from the still wet dispersion after coating the PU template. This is especially caused by the increased yield stress (7.5 Pa vs. 1.2 Pa). The share of closed strut porosity determined with the Archimedes method increased for samples from guar gum dispersion (especially for 40 vol% solid load samples) compared to samples with the same solid load of 40 vol% and other thickeners (methyl cellulose and wallpaper paste), as shown in Figure 4. This is in good agreement with the SEM micrograph of Figure 6a and images of µCT-measurement in Figure 8. Figure 5b [14]) shows the microstructure of a replica foam dried at ambient conditions, the thickening agent was guar gum, too. No lamellar material or lamellar pore spacings or bubble-shaped voids were found.

As shown in Figure 9, the dispersion coating behavior onto the PU template struts—SEM micrographs show the critical part, sharp edge of the PU foam strut—varies for different thickening agents. As a consequence, the compressive strength of foams was influenced, as it was shown for methylcellulose in ref. [50].

### 3.3. Compressive Strength

**Figure 10:**
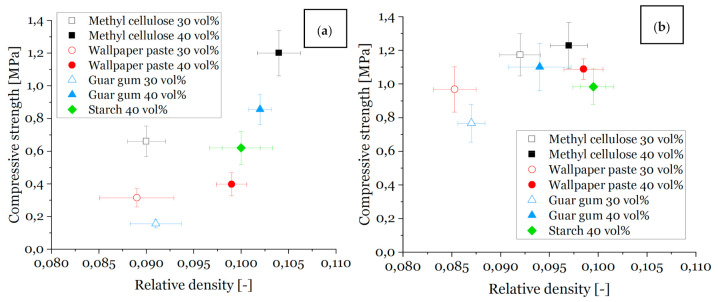
Compressive strength vs. relative density of all sample series and solid loads, sintered at 1650 °C; (**a**): samples with freeze casting, (**b**): samples dried at room temperature.

The compressive strength values are shown in Figure 10a for samples with pores from freeze-drying and in Figure 10b for samples dried at ambient conditions at room temperature (without additional pores from freeze-drying).

As shown in Figure 10, the compressive strength and the relative density of the samples increase with increasing solid content in the ceramic dispersion used for sample preparation, whereby the sample weight kept constant during template coating, independent of the solid content. Therefore, the fraction of ceramic particles within the wet dispersion coated on the template is higher for higher solid content dispersions and, as a consequence, the relative density of the foams made therefrom is higher as well [14].

The surface-to-volume ratio for the samples with additional pores due to freeze-drying is increased for all foams. The surface-to-volume ratio decreases for samples with higher solid content and is still slightly increased compared to samples that were dried at room temperature.

The values of relative density lie in a range of 8% to 11% for all samples and those values are similar for different foam series, e.g., for 30 vol% freeze-dried with thickener methyl cellulose foams, a total foam porosity ranging from 90.8% to 91.2% is observed, and for wallpaper paste foams with the same conditions, the porosity ranges from 90.7% to 91.5%. Hence, the compressive strength of these materials compares well.

Furthermore, the compressive strength is higher for a higher solid content [14] and for foams without freeze casting, as the number of defects in the strut material is lower [14,22]. The same reason counts for the highest compressive strength values found for the samples with methylcellulose as a thickener: the viscosity of the methylcellulose containing dispersion was the highest, cf. Figure 3, and gives therefore the best conditions for a homogeneous and nearly defect-free template coating, cf. also Figure 8. [50] The compressive strength for the foams made from the dispersions with methylcellulose and 30 vol% solid content are in the range of 0.3 MPa to 0.9 MPa. Consequently, this is an improvement compared with the strength results of, e.g., freeze-dried foams with guar gum as a thickener with the same solid load (0.1 MPa) [14]. Considering the Weibull moduli (Table 4), it is noticeable that the samples investigated in this article achieved values comparable to foams without additional pores and the thickener methyl cellulose [51] or increased solid content in the ceramic dispersion [62].

For a 40 vol% dispersion solid content, the compressive strength for samples from methylcellulose and guar gum is between 0.8 MPa and 1.3 MPa.

## 4. Conclusions

It was shown that the Schwartzwalder process for the manufacturing of open-cellular alumina foams in combination with freeze-drying of as-coated foam templates is feasible for a number of different thickening agents and for a variation of the alumina particle solid load in the coating dispersion. A solid load of 30 vol% leads to a higher total strut porosity as well as broader pore spacings and thinner material lamellae in the foam struts compared to a solid load of 40 vol%; these characteristics underwent only a small variation when different thickeners were used.

In spite of the high porosity and the lamellar structure of the foam struts, a compressive strength of up to 1.3 MPa at a total strut porosity of 46% and an overall total porosity between 90% and 91% was reached for foams processed with methyl cellulose as a thickener. The use of guar gum as a thickener, however, resulted in the formation of additional bubble-shaped pores, which, in turn, reduced the compressive strength and increased the share of the closed strut porosity (especially for higher solid loads). This work shows important basics regarding the combination of both methods (replica and freezing technique) and highlights their potential for usage as carriers for active materials.

## Figures and Tables

**Table 1:** Concentration of thickening agents in water.

Thickening Agent	Amount (g) in 25 mL Water
guar gum (G)	0.675
modified methyl cellulose (wallpaper paste) (WP)	1.5
methyl cellulose (MC)	1.5
potato starch (S)	3.125

**Table 2:** Characteristics of thickening agents used (after [31]).

Thickener	Source	Chemical Composition	Shear Stability	Gelation	Film Formation	Thickening Effect
guar gum	endosperm of seed	polysaccharide of D-mannose and D-galactose	shear thinning	no	low	high
starch	seed extracts (germs, roots)	α-D-glucose	low after gelation, irreversible viscosity loss	upon heating	high	high
methyl cellulose	wood pulp or cotton linters	linear polymer of β-D-glucose with (CH_3_)-substituents	shear-thinning with re-thickening effect (after rest-time), gels are very thixotropic	reversible gelation upon heating	high	low to high
wallpaper paste	mixture of starch and methyl cellulose					

**Table 3:** Dispersion compositions for polyurethane (PU) foam coating.

Dispersion Type	30 vol% Dispersion		40 vol% Dispersion	
Material	wt%	vol%	wt%	vol%
Al_2_O_3_ powder	61.64	29.08	70.88	38.42
distilled water and thickening agent according to Table 1	36.51	68.04	26.99	58.26
deflocculant Dolapix CE 64	0.92	1.44	1.06	1.66
binder Optapix PA 4G	0.92	1.44	1.06	1.66

**Table 4:** Weibull-modulus m (Visual-XSel 14.0) and object-surface-to-object-volume ratio of investigated samples.

Sample	MC 30 FC/RT	WP 30 FC/RT	GG 30 FC/RT	MC 40 FC/RT	WP40 FC/RT	GG 40 FC/RT	S 40 FC/RT
Weibull	3.511/4.967	2.544/3.07	2.76/3.313	6.087/4.185	2.885/8.610	5.258/3.833	3.38/4.653
R^2^	0.9806/0.9163	0.9299/0.9013	0.9407/0.9625	0.8870/0.9314	0.9258/0.9013	0.9357/0.9511	0.9445/0.9209
Obj. Surf./Obj. Vol.	70.8	51.5	69.6/26.3	27.8	30.2	48.3	

## Data Availability

All relevant data are mentioned in the publication.
